# Comparative proteomic profiles of *Aspergillus fumigatus *and *Aspergillus lentulus *strains by surface-enhanced laser desorption ionization time-of-flight mass spectrometry (SELDI-TOF-MS)

**DOI:** 10.1186/1471-2180-11-172

**Published:** 2011-07-28

**Authors:** Claudine Pinel, Marie Arlotto, Jean-Paul Issartel, François Berger, Hervé Pelloux, Renée Grillot, Françoise Symoens

**Affiliations:** 1 Institut de Neurosciences « Plateforme de Transcriptomique et de Protéomique Cliniques », (INSERM U836), Université Joseph Fourier, rue de la Chantourne, Grenoble, 38043, France; 2 Laboratoire de Parasitologie-Mycologie, Institut de Biologie et Pathologie (IBP), Centre Hospitalier Universitaire Albert Michallon, BP 217, Grenoble, 38043, France; 3 Mycology & Aerobiology Section, Scientific Institute of Public Health, 14 rue Juliette Wytsmanstreet, Brussels, 1050, Belgium

**Keywords:** *SELDI-TOF mass spectrometry*, *ProteinChips^®^*, *Aspergillus fumigatus*, *Aspergillus lentulus*, *proteomic analysis*

## Abstract

**Background:**

Surface-enhanced laser desorption ionization time-of-flight mass spectrometry (SELDI-TOF-MS) was applied to analyze the protein profiles in both somatic and metabolic extracts of *Aspergillus *species. The study was carried out on some *Aspergillus *species within the *Fumigati *section (*Aspergillus fumigatus *wild-types and natural abnormally pigmented mutants, and *Aspergillus lentulus*). The aim was to validate whether mass spectrometry protein profiles can be used as specific signatures to discriminate different *Aspergillus *species or even mutants within the same species.

**Results:**

The growth conditions and the SELDI-TOF parameters were determined to generate characteristic protein profiles of somatic and metabolic extracts of *Aspergillus fumigatus *strains using five different ProteinChips^®^, eight growth conditions combining two temperatures, two media and two oxygenation conditions. Nine strains were investigated: three wild-types and four natural abnormally pigmented mutant strains of *A. fumigatus *and two strains of *A. lentulus*. A total of 242 fungal extracts were prepared. The spectra obtained are protein signatures linked to the physiological states of fungal strains depending on culture conditions. The best resolutions were obtained using the chromatographic surfaces CM10, NP20 and H50 with fractions of fungi grown on modified Sabouraud medium at 37°C in static condition. Under these conditions, the SELDI-TOF analysis allowed *A. fumigatus *and *A. lentulus *strains to be grouped into distinct clusters.

**Conclusions:**

SELDI-TOF analysis distinguishes *A. fumigatus *from *A. lentulus *strains and moreover, permits separate clusters of natural abnormally pigmented *A. fumigatus *strains to be obtained. In addition, this methodology allowed us to point out fungal components specifically produced by a wild-type strain or natural mutants. It offers attractive potential for further studies of the *Aspergillus *biology or pathogenesis.

## Background

*Aspergillus *species comprise strains of medical and industrial importance. Only twenty out of the 250 species of the *Aspergillus *genus were isolated from patients with fungal diseases. Among them, A. *fumigatus *is the most important airborne fungal pathogen involved in various forms of aspergillosis in humans and animals [1-3]. Infections caused by this opportunistic and ubiquitous fungus can lead to fatal invasive aspergillosis in immunocompromised hosts with neutrophil deficiencies [4]. Its potential virulence is still poorly understood but it is probably associated with multiple and specific fungal factors, (among which its thermotolerance), in combination with host factors [5]. Recently, *A. lentulus *a species closely related to *A. fumigatus *within the *Fumigati *section, has been described by Balajee *et al*. [6]. This species has been associated with the same pathologies [7]. Moreover, it is naturally resistant to several antifungal drugs [8,9].

The availability of a sequenced and annoted genome of *A. fumigatus *provided a new starting point to understand the biology of this medically important fungus [10]. So far, few studies have been published about the proteomics and modification of protein expression under different environmental conditions. The techniques used are essentially based on two-dimensional electrophoresis (2DE) which allows the detection and then the purification of fungal compounds for further identification. However, even after optimization, this method is time-and sample-consuming [11,12]. More recently matrix-assisted laser desorption ionization time of flight mass spectrometry (MALDI-TOF-MS) which associates sensitivity and efficacy, has been applied to analyze the protein composition of fungal proteome [13-18]. This methodology proved useful for unambiguous identification of *Aspergillus *and *Penicillium *species [15,16]. Another mass spectrometry approach, the surface-enhanced laser desorption ionization time-of-flight mass spectrometry (SELDI-TOF-MS) has not yet been applied to detect fungal markers. This method provides specific advantages over conventional MALDI-TOF approaches as it combines chromatography on plane surfaces and mass spectrometry. SELDI-TOF-MS is specifically useful for comparative studies of selected components. The selective protein retention on the different target surfaces of the ProteinChips^® ^arrays allows the rapid analysis of complex mixtures. Since its first description [19], the SELDI-TOF-MS method has been widely used to find specific markers in cancerous, cardiovascular, neurological and infectious diseases [20-27]. The SELDI-TOF technology also proved successful to allow the identification of a post translational modified form of vimentin that discriminates infiltrative and non infiltrative meningiomas [28]. In microbiology, SELDI-TOF-MS was applied on *Acidithiobacillus ferrooxidans *[29] in order to better understand the physiological responses and biological adaptation of this pathogen to environmental conditions. In parasitological research, it was also applied to study the biological disturbances and detection of diagnostic markers in African trypanosomiasis [30,31].

The strategy of protein expression profiling allows the selection of proteins of interest or specific biomarkers and gives information on the best way to purify and further characterize them. Indeed, the best suited chromatographic material and the proper elution conditions to use for purification of the proteins of interest can be predicted from the binding behavior of the protein detected on the ProteinChip^® ^arrays. This technique like MALDI-TOF requires a minimal amount of proteins and is really appropriate for high throughput screening, particularly to distinguish up and down regulated proteins.

The aim of the present study, after selection of the culture conditions, was to assess the reliability of SELDI-TOF-MS method to analyze and discriminate crude fungal extracts (both somatic and metabolic fractions) of *A. fumigatus *and *A. lentulus*. It was also applied to discriminate natural abnormally pigmented mutant strains from a reference strain of *A. fumigatus *(strain used for annotation of the genome).

## Results and discussion

### Optimization of the SELDI-TOF parameters (ProteinChips^®^, amount of protein, storage of extracts, reproducibility)

Among the different ProteinChips^® ^tested: CM10, NP20, H50, Q10, IMAC30-Zn_2 _and IMAC30-Cu_2_, only CM10, NP20 and H50 chips were suitable. Binding of fungal components to the other ProteinChips^® ^was too weak to allow efficient profile analysis.

**Figure 1:**
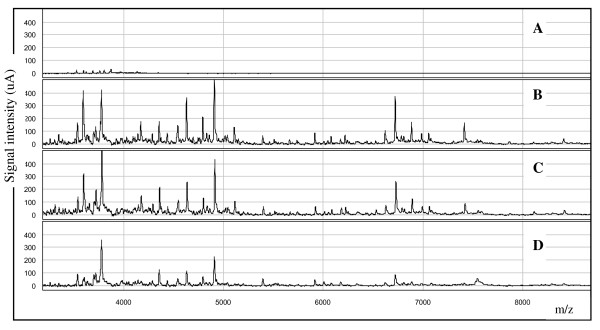
**SELDI-TOF spectra on CM10 ProteinChips^® ^of somatic extract of wild-type *A. fumigatus *(strain IHEM 22145) grown at 37°C on modified Czapeck medium**. (A) Profile of the negative control (medium without fungal culture); (B) Fungal extract analysed immediately after preparation; (C) Profile of the same fraction analysed, in the same conditions, after storage at -20°C for seven days; (D) Profile of the same extract analysed in the same conditions, after storage at 4°C for seven days.

The total amount of proteins spotted on the different ProteinChips^® ^giving the best peaks resolution was 5 μg on CM10 and H50 surfaces and 2 μg on NP20 chip. Each preparation was analysed in duplicate on the ProteinChips^®^. The spectra obtained from the culture media alone used as negative controls (concentrated modified Sabouraud and Czapeck media both without fungal cultures) did not interfere with the fungal protein spectra as the backgrounds were very low, few peaks of very low intensity were detected only under 4 kDa (Figure 1A).

Sample storage at -20°C did not alter the protein profiles (Figure 1B, C). However, as expected but never previously published to our knowledge for fungal extracts, the degradation was noticeable if the sample was stored at 4°C for seven days (Figure 1D). As numerous fungal proteins are proteolytic enzymes, the sample preparation and the storage conditions were of great importance in comparative studies. A strictly well defined protocol for the preparation and the rapid freezing at -20°C of the extracts are essential to preserve native proteins [32]. Due to the absence of protease inhibitors, proteolysis may occur during sample preparation. However, in the conditions used to preserve the fungal proteins, we argued that the possible degradation could be homogenous in all samples and altered slightly the comparative studies. The coefficient of variation of peak profiles on CM10 evaluated on three extracts from simultaneous cultures reached an average of 14.2%, lower reproducibility was obtained on NP20 (24.6%) and on H50 (35.4%).

### Selection of culture parameters: type of fractions, temperature, medium, oxygenation

In order to select the culture conditions giving an abundance of fungal components qualitatively detected on chromatographic ProteinChips^®^, we analyzed the somatic and metabolic protein patterns on NP20 and CM10 ProteinChips^® ^of the three wild-types strains *of A. fumigatus *(IHEM 9599, IHEM 18963 and IHEM 22145) using eight culture conditions (two temperatures: 25°C and 37°C, two oxygenation conditions: stationary and shaken culture, two media: modified Sabouraud and Czapek). Static and shaken fungal cultures were incubated at 37°C for four days and at 25°C for seven days.

#### Somatic and metabolic extracts

In the metabolic fractions, the total amount of proteins was at least three times as low as in the somatic fractions. Thus in the secretome (metabolic fractions), specific proteins in low abundance should be undetected in the mixture of the two types of extracts [33]. All fungal extracts from somatic and metabolic fractions obtained from the three wild-types strains of *A. fumigatus *were classified into two distinct clusters, whatever the growth conditions used (data not shown). As expected, this result highlights differences in protein profiles between these two types of extracts.

#### Temperature, oxygenation and medium

We observed great variations of protein patterns under various environmental conditions with the samples from the three wild-types strains of *A. fumigatus*. The number of significant differences (p < 0.05) in protein profiles according to growth conditions used were important depending on temperature. In our observations, these differences decreased with oxygenation and medium respectively.

#### Temperature

**Figure 2:**
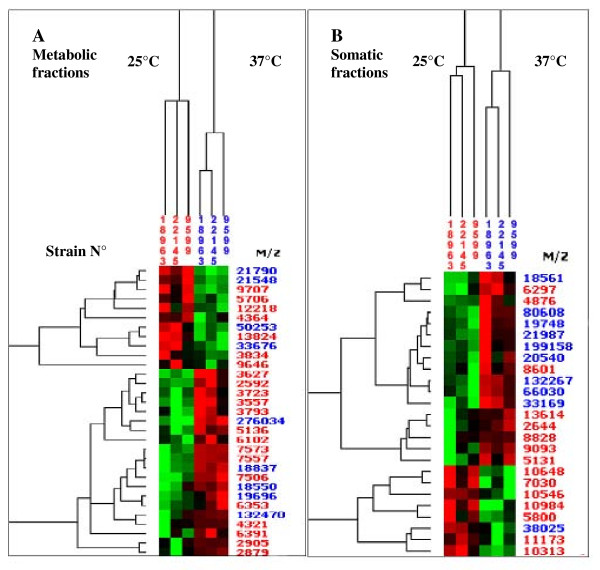
**Proteomic analysis of the temperature effects**. The hierarchical clustering obtained on CM10 ProteinChips^® ^with metabolic extracts (A) and somatic extracts (B) with the three wild-type *A. fumigatus *strains (IHEM 18963, IHEM 22145, IHEM 9599). The three extracts, one for each strain, obtained at 25°C (in red) and at 37°C (in blue) are indicated on the top of the figure. Values on the right indicate the molecular mass of protein differentially expressed according to the laser intensities used (in red 2000 nanoJoule (nJ) and in blue 4000 nJ). Two clusters were observed according to growth temperatures with the metabolic and the somatic extracts. Higher number of proteins was up regulated at 37°C than at 25°C in both fractions. In the dendrograms shown, the red, black or green colour indicates that the relative intensity of the protein concentration is respectively higher, intermediate or lower than the mean value.

The metabolic and somatic fractions from the three strains were separated into two distinct clusters according to growth temperature. Temperature modified the protein expressions in the same way for the three strains examined. Upregulated proteins were 60% higher at 37°C versus 25°C in both metabolic and somatic extracts (Figures 2A and 2B). In our conditions, twenty proteins were shown to be overexpressed at 37°C versus 25°C from the three wild-types strains of *A. fumigatus *strains. Protein overexpression at 37°C, also documented in our study, has already been pointed out. Some overexpressed proteins have been supposed to be involved in *A. fumigatus *virulence [34]. Essential genes for growth at 37°C have already been identified, and have been shown to play an important role in *A. fumigatus *survival and dissemination during invasive aspergillosis [35,36].

#### Oxygenation

On CM10 and NP20 ProteinChips^®^, two distinct clusters were obtained depending on oxygenation conditions for all the fungal samples analyzed whatever the temperature and media applied to growth conditions (data not shown).

Oxygen and a functional respiratory chain have been demonstrated to be essential for the germination process and mycelial development of *A. fumigatus *[37]. The protein patterns for both the metabolic and somatic fractions are notably influenced by oxygenation. From cultures with modified Sabouraud medium at 37°C, we observed 65 significant peaks out of 122 between static and shaken cultures for the somatic *A. fumigatus *extracts and 55 out of 112 for the metabolic fractions (p < 0.05) (data not shown). *Aspergillus fumigatus *is exposed to rapid changes in hypoxic conditions at sites of inflammation. The response to stressful conditions is likely to be an important virulence attribute of this pathogenic mold [5,38].

#### Medium

On modified Sabouraud medium the number of upregulated proteins was higher than in the modified Czapeck medium for the three wild-types strains of *A. fumigatus*. The medium composition obviously acts on fungal growth. The medium influence has already been shown using 2-D electrophoresis for *A. fumigatus *[12] and MALDI-TOF analysis for *A. oryzae *[39].

In conclusion, the results obtained clearly show that *A. fumigatus *proteome is dynamic and will adapt to its immediate environment as described for *Aspergillus nidulans *[40] and bacteria [41]. The three strains of *A. fumigatus *responded in the same way according to the variations of environmental factors such as temperature, medium and oxygenation. For comparative analysis applied to discriminate strains and species, the modified Sabouraud medium and incubation temperature at 37°C were selected.

### Comparison of atypical pigmented *A. fumigatus *strains versus a wild reference strain

Fewer fungal proteins were retained on H50 ProteinChips^®^. As pigmented structures and fungal surface layer consist mainly of hydrophobic proteins [42], H50 ProteinChip^® ^was chosen in association with CM10 to compare the profiles obtained from one reference wild-type strain of *A. fumigatus *(IHEM 18963/Af 293) and four abnormally pigmented strains: three white strains (IHEM 2508, IHEM 9860 and IHEM 13262) and one brown strain (IHEM 15998). Fungal extracts were obtained from three sets of cultures started simultaneously and one set started another day. These cultures were performed on modified Sabouraud medium at 37°C. Since pigments are produced during conidia formation (static culture), we maintained the two oxygenation conditions allowing the analysis of proteins from hyphae and conidia (static culture) and from hyphae (shaken culture).

A previous study on these strains [42] has shown that for two of the three white mutants investigated, the *ALB1 *gene involved early in the melanin synthesis steps has mutated. For the brown mutant, a point mutation in the *ARP2 *gene involved in a later step of the melanin synthesis has been observed. These three strains presented white or brown powdery colonies. For the strain IHEM 13262, we observed poor conidiation and velvety colonies.

As previously observed with the three wild-type strains, the software classified 100% of the metabolic and somatic samples into two clusters in function of oxygenation conditions with the two types of ProteinChips^® ^used (CM10 and H50).

**Figure 3:**
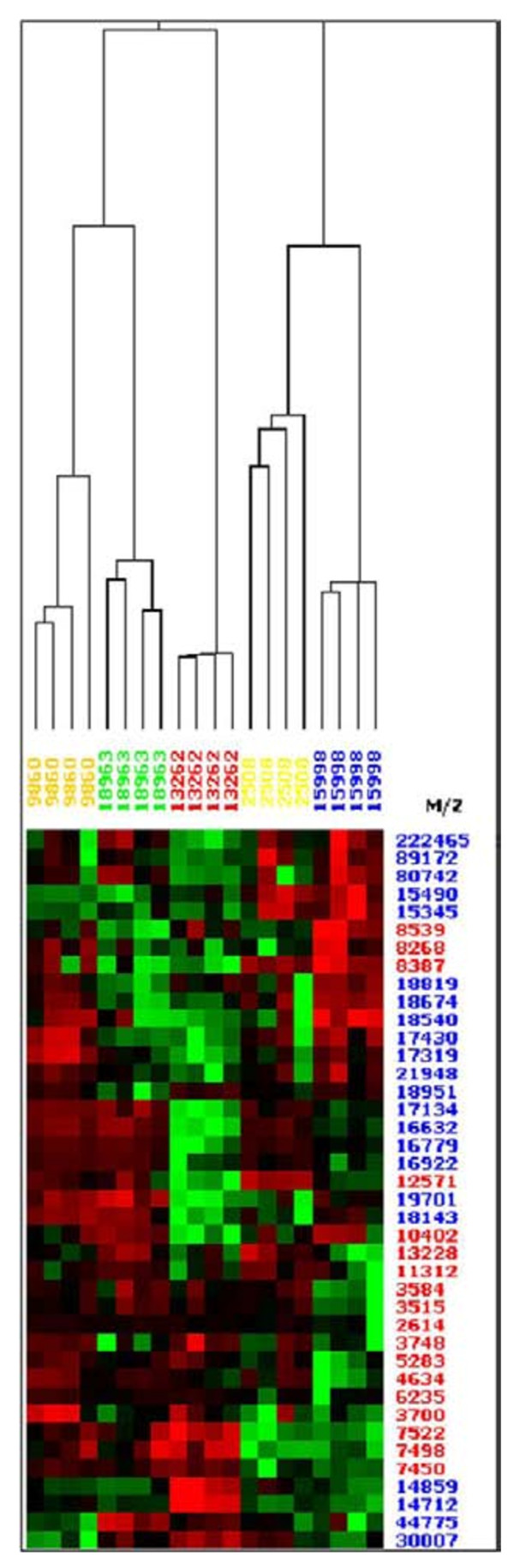
**Proteomic comparison between abnormally pigmented strains and a wild-type reference strain of *A. fumigatus *on CM10 ProteinChips^®^**. Hierarchical classification of metabolic extracts obtained in static culture for the five strains grown on modified Sabouraud medium at 37°C: white M IHEM 9860 (orange), reference WT strain IHEM 18963 (green), white M IHEM 13262 (red), white M IHEM 2508 (yellow) and brown M IHEM 15998 (blue). The proteins differentially expressed (p < 0.05) were listed on the right of the figure with laser intensities of 2500 nJ (in red) and of 4500 nJ (in blue).

Furthermore, the SELDI-TOF-MS analysis of metabolic extracts obtained from static cultures performed on CM10 and on H50 ProteinChips^® ^resulted in the classification of the five *A. fumigatus *strains (wild-types and mutants) in five clusters. Figure 3 illustrates the discrimination of the metabolic fractions obtained in static culture from the five strains on CM10 ProteinChip^®^. Using this ProteinChip^® ^with the five strains under study, eighteen proteins obtained from the metabolic fractions (shaken and static cultures) and thirteen from the somatic extracts (shaken and static cultures) expressed differently (p < 0.05). Some of them were specifically found in the extracts from the wild-type strain in the metabolic and in somatic fractions. On H50 surfaces, only twelve proteins expressed in significantly different ways in the 2 types of extracts.

**Figure 4:**
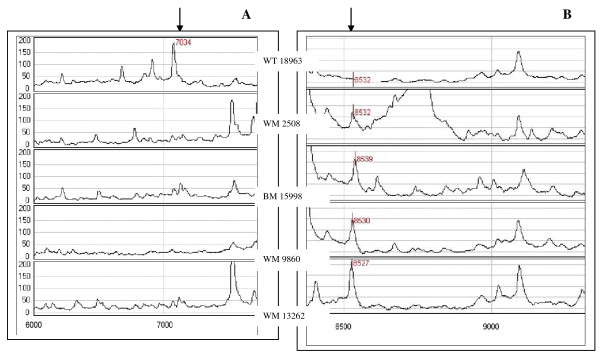
**Examples of SELDI-TOF spectra of differentially expressed proteins on CM10 ProteinChips^®^**. A: The protein profile showed a protein of 7034 Da mostly expressed by the wild-type strain in the somatic fraction obtained from shaken culture, B: A peak around 8530 Da was detected only in the metabolic fractions obtained from static cultures of the four abnormally pigmented *A. fumigatus *strains (IHEM 2508, 15998, 9860 and 13262). WT: wild-type, WM: White mutant, BM: Brown mutant.

An example of differentially expressed proteins found is illustrated in Figures 4A and 4B with the CM10 ProteinChips^®^. A protein with a molecular mass of around 7000 Da was detected in the somatic extracts from the wild-type strain with both ProteinChips^® ^used (p < 0.021) but not in the extracts obtained from the four abnormally pigmented *A. fumigatus *strains (Figure 4 A). On the contrary, a protein with a molecular mass of around 8530 Da was found to be secreted by all four mutants in metabolic fractions from static cultures where pigment and conidia were developed (p < 0.039) but was not detected in metabolic fractions obtained from the wild-type strain as shown in Figure 4B. Its relation to pigmentation or induction or repression of other genes remains to be established.

The SELDI-TOF comparison of these four natural mutants with the wild-type reference strain is powerful. This analysis indicated protein masses of interest which could open further investigations in the comparative study between mutants and wild-type strains. As observed, this method is highly suitable to separate low molecular weight compounds and could provide complementary data to other analytical techniques [43]. Thus, as described for bacteria [25], this method may be also suitable to discriminate isolates within the same species.

### Comparison of *A. fumigatus *and *A. lentulus *extracts

**Figure 5:**
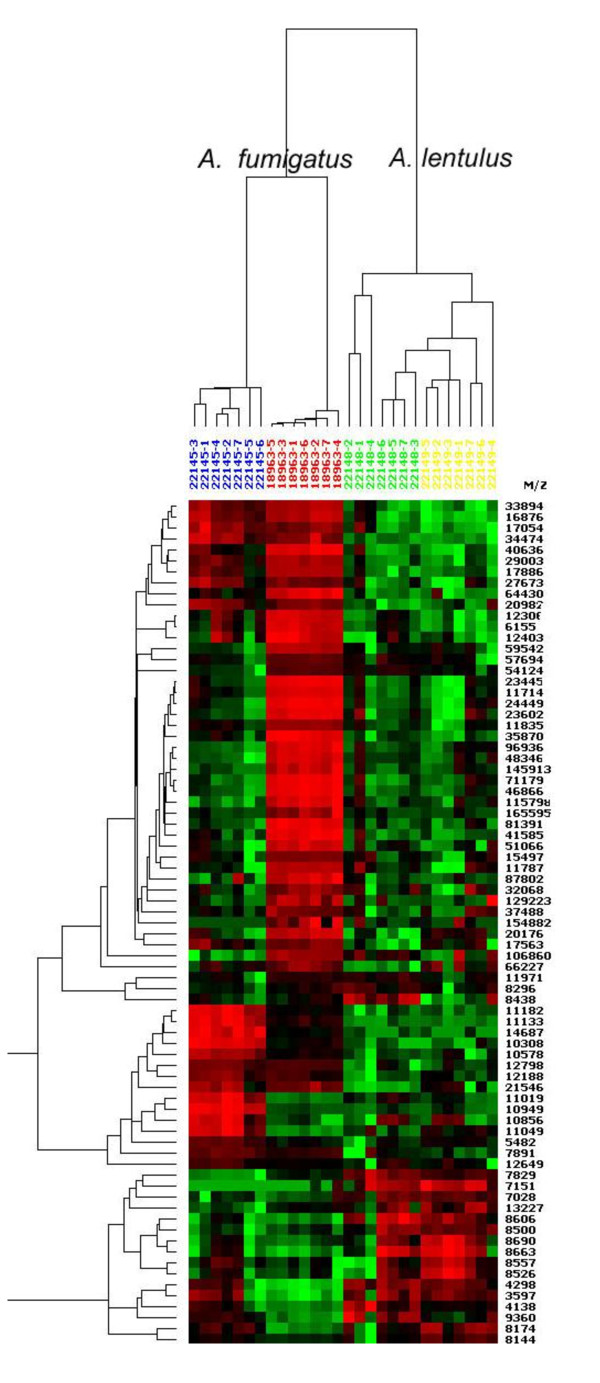
**The hierarchical clustering of *A. fumigatus *and *A. lentulus *metabolic extracts**. (shaken culture, modified Sabouraud medium at 37°C, on CM10 ProteinChips^®^) showing 77 out of 101 significant p values (p < 0.01). From the right to the left: in red and blue colour *A. fumigatus *(strains IHEM 22145 and IHEM18963) and in green and yellow colour *A. lentulus *(strains IHEM 22148 and IHEM 22149).

In addition to the separation of strains within the same species, we applied hierarchical clustering to differentiate *A. fumigatus *from *A. lentulus*, a closely related species from the *Fumigati *section, using CM10 and NP20 ProteinChips^® ^chosen for to their good reproducibility. Metabolic extracts (from seven different sets of experiments: six grown simultaneously and one independently) from *A. fumigatus *and *A. lentulus *strains were classified into distinct clusters on CM10 (Figure 5) as well as on NP20 ProteinChips^® ^(not shown). Ten out of 101 proteins showed over expression only in the *A. fumigatus *extracts (Figure 5). Somatic extracts from the two *Aspergillus *species were also separated into two distinct clusters according to the species. However, the somatic extracts from the two *A. lentulus *strains were not completely separated (not shown). The best resolution was obtained with the metabolic samples on CM10 ProteinChip^®^, perfect distinction was obtained between the two species and between the two isolates within the same species (Figure 5).

Even if these two species are morphologically very similar, it has been shown that they display differences in their cell wall composition, i.e. *A. lentulus *contains less chitin than *A. fumigatus *[9], is less thermotolerant and produced different secondary metabolites. The conidium surface is smooth and lack hydrophobic rodlet layer. These biochemical and structural differences could explain a distinguishable protein pattern.

## Conclusions

The qualitative and quantitative results provided by SELDI-TOF-MS can be obtained in a rapid, sensitive and reproducible way if careful and standardized procedures are used for sample preparation and storage. The spectra obtained on CM10 chip essentially are protein signatures representative of the strains and of their physiological states. The proteomic analysis allows the distinction of not only the closely related species *A. fumigatus *and *A. lentulus *but also natural mutants within the *A. fumigatus *species. Furthermore, it could be an analytical tool in the research of molecular mechanisms involved in the physiopathology of *A. fumigatus*. It could be also a powerful method for quality control of antigenic extracts for diagnosis purposes.

## Methods

### Fungal strains

All the strains detailed in Table 1 were referenced and preserved in the BCCM/IHEM Collection of the Scientific Institute of Public Health, Brussels, Belgium (http://bccm.belspo.be/db/ihem_search_form.php). They consisted of three wild-type strains of *A. fumigatus *(WT), including strain Af 293 used for genome sequencing of *A. fumigatus *and four natural abnormally pigmented strains of *A. fumigatus *(M) among which one brown and three white strains. All the isolates were identified by macroscopic and microscopic morphology. Their identification was confirmed by internal transcribed spacers (ITS) regions of ribosomal DNA gene and by β-tubulin gene sequencing [8,44]. Two *A. lentulus *strains came from the CBS collection (Centraalbureau voor Schimmelcultures, Utrecht, The Netherlands).

**Table 1:** References, characteristics and origin of the different *Aspergillus fumigatus *(*Afu*) and *Aspergillus lentulus *(*Ale*) strains used

IHEM Number	Other acronym	Species *Afu/Ale*	Strain characteristics	Substrate origin, underlying disease	**Year isolation**, Country
9599		*Afu*	WT*	human blood culture, IA (hepatoblastoma),	1995, France
22145		*Afu*	WT	Human cerebral biopsy, IA (leukaemia)	2001, France
18963	Af293	*Afu*	WT	Human lung, IA (autopsy), reference sequencing project	1993, UK
2508		*Afu*	White M**	Hospital environment	1985, Belgium
9860	CBS 386.75	*Afu*	White M	Usar soil	1975, India
13262	CBS 110.46 = ATCC 16907	*Afu*	White M	Type strain of helvola mutant	1939
15998		*Afu*	Brown M	Human sputum (cystic fibrosis)	1999, France
22148	CBS 175.97	*Ale*		Dolfin nostril	1996, The Netherlands
22149	CBS 116883	*Ale*		Soil	2003, Korea

*WT: wild-type, **M: mutant, IA: invasive aspergillosis.

### Culture conditions

In order to optimize the growth condition for the characterization of protein extracts from *A. fumigatus*, eight culture conditions were selected: two temperatures corresponding to those used for sample cultures in medical mycology (25°C and 37°C), two media (modified Sabouraud and modified Czapeck), and two oxygenation conditions (static and shaken cultures). Modified Sabouraud medium consisted of dextrose 20 g/l, neopeptone 10 g/l, MgSO_4 _0.5 g/l, KH_2_PO_4 _0.5 g/l, oligoelements solution 1 ml of the following solution: H_3_BO_3 _58 mg/l, CuCl_2_. 2H_2_O 270 mg/l, MnCl_2_.4H_2_O 78 mg/l, ZnCl_2 _4.2 mg/l, FeCl_2_.4H_2_O 3 mg/l, (NH_4_)_6_Mo_7_O_24_.4H_2_O 0.2%. Modified Czapek medium consisted of saccharose 15 g/l, yeast nitrogen base 1 g/l, brain heart 1 g/l, NaNO_3 _3 g/l, K_2_HPO_4 _1 g/l, KCl 0.5 g/l, MgSO_4 _0.5 g/l, FeSO_4_.7H_2_O 0.01 g/l). Both media were home-made. The strains were grown at 25°C for seven days and at 37°C for four days. The oxygenation conditions corresponded to static culture (Roux Flasks) and to shaken culture (gyratory shaker at 150 rpm).

### Preparation of fungal protein extracts

Fungal mycelium and conidia were collected from Roux flask and filtered on a folded Whatman filter (Schleicher & Schuell 10311853). Shaken cultures were also filtered in the same conditions to separate growth medium from mycelium.

Somatic proteins were mechanically extracted from the fungus mycelium with Ultraturrax in NH_4_HCO_3 _buffer 0.4%, shaken overnight at 4°C and centrifuged at 10 000 g. The supernatant was concentrated with Amicon Ultra UFC900324 (Millipore, USA). The amount of protein was estimated by colorimetry (Biophotometer Eppendorf) using QuickStart Bradford Dye Reagent (Bio-Rad protein assay 500-0205) with Bovine Serum Albumin as standard (Bio-Rad 500-026). The average of protein fraction in the extracts was 60% to 70% (wt/wt).

The metabolic extracts were directly concentrated from the culture medium with Amicon Ultra. The extracts were freeze dried for long-term stability (freeze dryer Christ Epsilon 1D, Germany).

In order to assess the variability of the protein expression, the extracts from the strains listed in Table 1 were prepared from three cultures performed simultaneously and from two to four cultures performed at different days.

### SELDI-TOF-MS analysis

To analyze the fungal spectra using SELDI-TOF-MS, the extracts were applied to weak cation exchange (CM10), normal silicate surface (NP20), reverse phase (H50), strong anion exchange (Q10) and immobilized metal affinity capture (IMAC30-Cu_2 _or IMAC30-Zn_2_) ProteinChips^® ^in 96-sample bioprocessors (Bio-Rad Laboratories, Hercules, CA, USA). All these surfaces were tested in order to select those retaining a large number of fungal compounds with a good resolution. The ProteinChips^® ^preparation, samples and matrix application were performed according to the manufacturer's instructions with slight modifications described below.

The freeze-dried samples were diluted with sterile distilled water in order to obtain 1 μg of total protein/μL. To preserve proteins from enzymatic degradation, the dilutions were immediately stored at -20°C until use. Five μg of sample were first diluted (1/20) in binding buffer and loaded on CM10, Q10, H50 and IMAC30-Cu_2 _or IMAC30-Zn_2 _ProteinChip then incubated for 1hr at room temperature. The unbound proteins were removed by washing three times with 200 μL of the same buffer, the ProteinChips^® ^were quickly rinsed with pure water and left to dry. For NP20 ProteinChips^® ^, 2 μL of sample were applied on the spot and left to dry, and then washed three times with 5 μL of water. Matrix (100% saturated solution of sinapinic acid in 0.5% trifluoroacetic acid/50% acetonitrile) was applied to each spot (twice 0.8 μL). The absorbed proteins were then analyzed on a ProteinChip Reader (series 4000, Bio-Rad Laboratories, Hercules, CA, USA). Spectra were obtained using two different acquisition protocols, for low (2.5-14 kDa) and high (14-400 kDa) molecular mass proteins, respectively. External mass calibration was performed with ProteinChip All-in-One Protein Standard II (Bio-Rad, laboratories, Hercules, CA, USA). Peak annotation was performed after base-line subtraction, noise calculation, and normalization by total ion current (TIC). Peak detection was achieved with ProteinChip Data Manager Software and only peaks with a signal-to-noise ratio > 5 were used for analysis (Bio-Rad Laboratories, Hercules, CA, USA).

### Statistical analysis

Statistical analyses were performed using ProteinChip Data Manager 3.0 software (Bio-Rad Laboratories, Hercules, CA, USA). All the spectra were compiled, and qualified mass peaks (signal-to-noise ratio > 5) with mass-to-charge ratio (m/z) between 2.5 kDa and 250 kDa were auto detected. P-values were calculated using non parametric Mann-Whitney *U*-test, which tests the null hypothesis that the medians of the peak intensities of the groups are equal. A p-value less than 0.05 was accepted as statistically significant. The difference was also examined by hierarchical clustering.

## Competing interests

The authors declare that they have no competing interests.

## Authors' contributions

CP conceived the project and drafted the manuscript, MA processed the samples and analysed SELDI-TOF data, JPI, RG, FB and HP were involved in interpretation and presentation of the results, FS prepared the *Aspergillus *samples and co-directed the project. All authors read, discussed and approved the final manuscript.

## References

[B1] Latgé JP *Aspergillus fumigatus *and aspergillosis Clin Microbiol Rev 1999 12 310 350 10194462 10.1128/cmr.12.2.310 PMC88920

[B2] Latgé JP The pathobiology of *Aspergillus fumigatus* Trends Microbiol 2001 9 382 389 10.1016/S0966-842X(01)02104-7 11514221

[B3] Geiser DM Klich MA Frisvad JC Peterson SW Varga J Samson RA The current status of species recognition and identification of *Aspergillus* Stud Mycol 2007 59 1 10 10.3114/sim.2007.59.01 18490947 PMC2275194

[B4] Hohl TB Feldmesser M *Aspergillus fumigatus*: principles of pathogenesis and host defense Eukaryotic Cell 2007 6 1953 1963 10.1128/EC.00274-07 17890370 PMC2168400

[B5] Abad A Fernandez-Molina JV Bikandi J Ramirez A Margareto J Sendino J Hernando FL Ponton J Garaizar J Rementeria A What makes *Aspergillus fumig*atus a successful pathogen? Genes and molecules involved in invasive aspergillosis Rev Iberoam Micol 2010 27 155 182 10.1016/j.riam.2010.10.003 20974273

[B6] Balajee SA Gribskov JL Hanley E Nickle D Marr KA *Aspergillus lentulus *sp. nov., a new sibling species of *A. fumigatus* Eukaryot Cell 2005 4 625 632 10.1128/EC.4.3.625-632.2005 15755924 PMC1087803

[B7] Montenegro G Puch S Sanchez VM Jewtuchowicz MV Pinoni MV Relloso S Temporitti E Iovannatti CA Mujica MT Phenotypic and genotypic characterization of *Aspergillus lentulus *and *Aspergillus fumigatus *isolates in a patient with probable invasive aspergillosis J Med Microbiol 2009 58 391 395 10.1099/jmm.0.005942-0 19208894

[B8] Balajee SA Nickle D Varga J Marr KA Molecular studies reveal frequent misidentification of *Aspergillus fumigatus *by morphotyping Eukaryot Cell 2006 5 1705 1712 10.1128/EC.00162-06 17030996 PMC1595351

[B9] Staab JF Kahn JN Marr KA Differential *Aspergillus lentulus *echinocandin susceptibilities are Fksp-independent Antimicrob Agents Chemother 2010 54 4992 4998 10.1128/AAC.00774-10 20855747 PMC2981225

[B10] Nierman WC Pain A Anderson MJ Wortman JR Kim HS Arroyo J Berriman M Abe K Archer DB Bermejo C Bennett J Bowyer P Chen D Collins M Coulsen R Davies R Dyer PS Farman M Fedorova N Fedorova N Feldblyum TV Fischer R Fosker N Fraser A García JL García MJ Goble A Goldman GH Gomi K Griffith-Jones S Gwilliam R Genomic sequences of the pathogenic and allergenic filamentous fungus *Aspergillus fumigatus* Nature 2005 438 1151 1156 10.1038/nature04332 16372009

[B11] Shevchenko A Jensen ON Podtelejniko AV Sagliocco F Wilm M Vorm O Mortensen P Boucherie H Mann M Linking genome and proteome by mass spectrometry: large scale identification of yeast proteins from two dimensional gels Proc Natl Acad Sci USA 1996 93 14440 14445 10.1073/pnas.93.25.14440 8962070 PMC26151

[B12] Kniemeyer O Lessing F Scheibner O Hertweck C Brakhage AA Optimisation of a 2D electrophoresis protocol for the human pathogenic fungus *Aspergillus fumigatus* Curr Genet 2006 49 178 189 10.1007/s00294-005-0047-9 16362819

[B13] Grinyer J McKay M Nevalainem H Herbert BR Fungal proteomics: initial mapping of biological control strain *Trichoderma harzianum* Curr Genet 2004 45 163 169 10.1007/s00294-003-0474-4 14685766

[B14] Kim Y Nandakumar MP Marten MR Proteomics of filamentous fungi Trends in Biotechnology 2007 25 395 400 10.1016/j.tibtech.2007.07.008 17681627

[B15] Hettick JM Green BJ Buskirk AD Kashon ML Slaven JE Janotka E Blachere FM Schelchel D Beezhold DH Discrimination of *Aspergillus *isolates at the species and strain level by matrix-assisted laser desorption/ionization time-of-flight mass spectrometry fingerprinting Anal Biochem 2008 380 276 281 10.1016/j.ab.2008.05.051 18577370

[B16] Hettick JM Green BJ Buskirk AD Kashon ML Slaven JE Janotka E Blachere FM Schmechel D Beezhold DH Discrimination of *Penicillium *isolates by matrix-assisted laser desorption/ionization time-of-flight mass spectrometry fingerprintingy Rapid Commun Mass Spectrom 2008 22 2555 2560 10.1002/rcm.3649 18646251

[B17] Kim Y Nandakumar MP Marten MR The state of proteome profiling in the fungus genus *Aspergillus* Brief Funct Genomic Proteomic 2008 7 780 783 10.1093/bfgp/elm031 18187513

[B18] Marinach-Patrice C Fekkar A Atanasova R Gomes J Djamdjian L Brossas JY Meyer I Buffet P Snounbou G Datry A Hennequin C Golmard JL Mazier D Rapid species diagnosis for invasive candidiasis using mass spectrometry PloS One 2010 5 e8862 10.1371/journal.pone.0008862 20111603 PMC2810333

[B19] Hutchens TW Yip TT New desorption strategies for the mass spectrometry analysis of macromolecules Rapid Commun Mass Spectrom 1993 7 576 580 10.1002/rcm.1290070703

[B20] Seibert V Wiesner A Buschmann T Meuer J Surface-enhanced laser desorption ionization time-of-flight mass spectrometry (SELDI TOF-MS) and ProteinChip technology in proteomic research Pathol Res Pract 2004 200 83 94 10.1016/j.prp.2004.01.010 15237917

[B21] Tang N Tornatore P Weinberger SR Current developments in SELDI affinity technology Mass Spectrometry Rev 2004 23 34 44 10.1002/mas.10066 14625891

[B22] Poon TC Hui AY Chan HL Ang IL Chow SM Wong N Sung JJ Prediction of liver fibrosis and cirrhosis in chronic hepatitis B infection by serum proteomic fingerprinting: a pilot study Clin Chem 2005 51 328 335 10.1373/clinchem.2004.041764 15590748

[B23] Engwegen JY Gast A Schellens JH Beijnen JH Clinical proteomics: searching for better tumour markers with SELDI-TOF mass spectrometry Trends Pharmacol Sci 2006 27 251 259 10.1016/j.tips.2006.03.003 16600386

[B24] Abromovitz M Leyland-Jones B A system approach to clinical oncology: focus on breast cancer Proteome Sci 2006 4 1 15 10.1186/1477-5956-4-1 16595007 PMC1456950

[B25] Seibold E Bogumil R Vorderwürlbecke S Al Dahouk S Buckendhahl A Tomaso H Splettstoesser W Optimized application of surface-enhanced laser desorption/ionization time-of-flight MS to differentiate *Francisella tularensis *at the level of subspecies and individual strains FEMS Immunol Med Microbiol 2007 49 364 373 10.1111/j.1574-695X.2007.00216.x 17378900

[B26] Gupta P Lee KH Genomics and proteomics in process development opportunities and challenges Trends Biotechnol 2007 25 324 330 10.1016/j.tibtech.2007.04.005 17475353

[B27] Hodgetts A Levin M Kroll JS Langforgd PR Biomarker discovery in infections diseases using SELDI Future Microbiol 2007 2 35 49 10.2217/17460913.2.1.35 17661674

[B28] Bouamrani A Ramus C Gay E Pelletier L Cubizolles M Brugière S Wion D Berger F Issartel JP Increased phosphorylation of vimentin in non-infiltrative meningiomas PLoS One 2010 16 5e9238 10.1371/journal.pone.0009238 PMC2821924 20169076

[B29] He Z Zhong H Hu Y Xiao S Xu J Analysis of differential protein expression in *Acidithiobacillus ferrooxidans *grown under different energy resources respectively using SELDI-proteinChip technologies J Microbiol Meth 2006 65 10 20 10.1016/j.mimet.2005.06.006 16112213

[B30] Stiles JK Whittaker J Sarfo BY Thompson WE Powell MD Bond VC Trypanosome apoptotic factors mediates apoptosis in human brain vascular endothelial cells Mol Biochem Parasitol 2004 133 229 240 10.1016/j.molbiopara.2003.10.011 14698435

[B31] Agranoff D Stich A Abel P Krishna S Proteomic fingerprinting for the diagnosis of human African trypanosomiasis Trends Parasitol 2005 21 154 156 10.1016/j.pt.2005.02.003 15780834

[B32] Monod M Jousson O Utz R JP Latgé, WJ Steinbach *Aspergillus fumigatus *secreted proteases Aspergillus fumigatus and Aspergillosis 2009 ASM Press 87 106

[B33] Hogan DA Talking to themselves: autoregulation and quorum sensing in fungi Eukaryot Cell 2006 5 613 619 10.1128/EC.5.4.613-619.2006 16607008 PMC1459667

[B34] Bhabhra R Miley MD Mylonakis E Boettner D Fortwendel J Panepinto JC Postow M Rhodes JC Askew DS Disruption of the *Aspergillus fumigatus *gene encoding nucleolar protein CgrA impairs thermotolerant growth and reduces virulence Infect Immun 2004 72 4731 4740 10.1128/IAI.72.8.4731-4740.2004 15271935 PMC470587

[B35] Shankar J Nigam S Saxena S Madan T Sarma PU Identification and assignment of function to the genes of *Aspergillus fumigatus *expressed at 37°C J Eukaryot Microbiol 2004 51 428 432 10.1111/j.1550-7408.2004.tb00390.x 15352325

[B36] Askew DS *Aspergillus *virulence genes in a street-smart mold Cur Opin Microbiol 2008 11 331 337 10.1016/j.mib.2008.05.009 PMC2559812 18579432

[B37] Taubitz A Bauer B Heeseman J Ebel F Role of respiration in the germination process of the pathogenic mould *Aspergillus fumigatus* Curr Microbiol 2007 54 354 360 10.1007/s00284-006-0413-y 17486408

[B38] Willger SD Puttikamonkul S Kim SH Burritt JB Grahl N Metzler LJ Barbuch R Bard M Laurence CB Cramer RA A sterol-regulatory element binding protein is required for cell polarity, hypoxia adaptation, azole drug resistance and virulence in *Aspergillus fumigatus* PloS Pathogens 2008 4 e1000200 10.1371/journal.ppat.1000200 18989462 PMC2572145

[B39] Oda K Kakizono D Yamada O Iefuji H Akita O Iwashita K Proteomic analysis of extracellular proteins from *Aspergillus oryzae *grown under submerged and solid state culture conditions Appl Environ Microbiol 2006 72 3448 3457 10.1128/AEM.72.5.3448-3457.2006 16672490 PMC1472361

[B40] Kim Y Nandakumar MP Marten MR Proteome map of *Aspergillus nidulans *during osmoadaptation Fungal Genet Biol 2007 44 886 895 10.1016/j.fgb.2006.12.001 17258477

[B41] Egan S Lanigan M Shiell B Beddome G Stewart D Vaughan J Michalski WP The recovery of *Mycobacterium avium *subspecies *paratuberculosis *from the intestine of infected ruminants for proteomic evaluation J Microbiol Meth 2008 75 29 39 10.1016/j.mimet.2008.04.008 18547663

[B42] Pihet M Vandeputte P Tronchin G Renier G Saulnier P Georgeault S Mallet R Chabasse D Symoens F Bouchara JP Melanin is an essential component for the integrity of the cell wall of *Aspergillus fumigatus *conidia BMC Microbiol 2009 9 177 10.1186/1471-2180-9-177 19703288 PMC2740851

[B43] Kiehntopf M Siegmund R Deufel T Use of SELDI-TOF mass spectrometry for identification of new biomarkers: potential and limitations Clin Chem Lab Med 2007 45 1435 1449 10.1515/CCLM.2007.351 17970700

[B44] Leaw SN Chang HC Sun HF Barton R Bouchara JP Chang TC Identification of medically important yeast species by sequence analysis of internal transcribed spacer regions J Clin Microbiol 2006 44 693 699 10.1128/JCM.44.3.693-699.2006 16517841 PMC1393093

